# Fulminant encephalopathy in a child with hyperferritinemic sepsis: a case report

**DOI:** 10.1186/s12883-020-01661-z

**Published:** 2020-03-02

**Authors:** Lilin Huang, Shumei Peng, Ronghan Li, Danyu Xie, Dongping Huang

**Affiliations:** Department of Pediatrics, Guangdong Women and Children Hospital, Guangzhou, Guangdong China

**Keywords:** Fulminant, Sepsis-associated encephalopathy, Hyperferritinemic sepsis, Diffuse white matter abnormalities

## Abstract

**Background:**

Sepsis-associated encephalopathy (SAE) is epidemic in intensive care units and recognized as a fatal complication of sepsis. SAE is characterized by diffuse brain dysfunction and the correct diagnosis of SAE requires ruling out direct central nervous system (CNS) infection or other types of encephalopathy, such as hepatic encephalopathy, pulmonary encephalopathy, and other encephalopathy.

**Case presentation:**

We reported a rare case of a 5-year-old girl who presented with abdominal pain, vomiting, recurrent seizures, and coma. Brain magnetic resonance imaging (MRI) showed diffuse white matter abnormalities in the brain on day 1. Cerebrospinal fluid (CSF) tests revealed that protein levels and glucose levels were normal without pleocytosis. CSF PCRs for pathogens were all negative. The electroencephalography examination demonstrated diffuse, generalized and slow background activity. The patient showed the symptom of hyperferritinemic sepsis with multiple organ dysfunction syndrome (MODS). SAE was also diagnosed by ruling out other encephalitis or encephalopathy. The patient made marked improvements of clinical symptoms and the lesions on brain imaging disappeared completely within two months after appropriate treatment including antibiotic treatments, methylprednisolone, intravenous immunoglobulin, membrane-based therapeutic plasma exchange (TPE), and continuous renal replacement therapy (CRRT).

**Conclusions:**

SAE can be a fatal complication of sepsis which asks for immediate diagnosis and treatment. Few reports have focus on MRI imaging findings on the early onset of hyperferritinemic sepsis with MODS since these children were too ill to undergo an MRI scan. However, SAE might appear before other systemic features of sepsis are obvious, and MRI could show abnormal lesion in the brain during the early course. Therefore, MRI should be performed early to diagnose this fatal complication which would play important roles in improving the clinical outcomes by early initiation with appropriate treatments.

## Background

Sepsis-associated encephalopathy (SAE) is epidemic in intensive care units and has received a large amount of attention [1]. The correct diagnosis of SAE requires ruling out direct central nervous system (CNS) infection or other types of encephalopathy (such as hepatic encephalopathy, pulmonary encephalopathy, and other encephalopathy) [2]. There is no consensus about specific and effective treatments for SAE, as how its pathogenesis occurs is still unclear. There are few pediatric cases of SAE reported, and all had long stays in the ICU with poor prognoses. Herein, we report that a child with severe hyperferritinemic SAE was successfully treated within 2 months. To our knowledge, this is the first study describing brain magnetic resonance imaging (MRI) of a patient with hyperferritinemic sepsis on the day one after the onset of symptoms since these children are often too ill to undergo an MRI scan upon presentation. The fulminant clinical features and extensive brain lesions throughout the white matter suggested that the patient was in an extremely dangerous state. However, the patient in our case recovered quickly, and the lesions on brain imaging were reversed completely upon accurate diagnosis and effective treatment.

## Case presentation

**Fig. 1:**
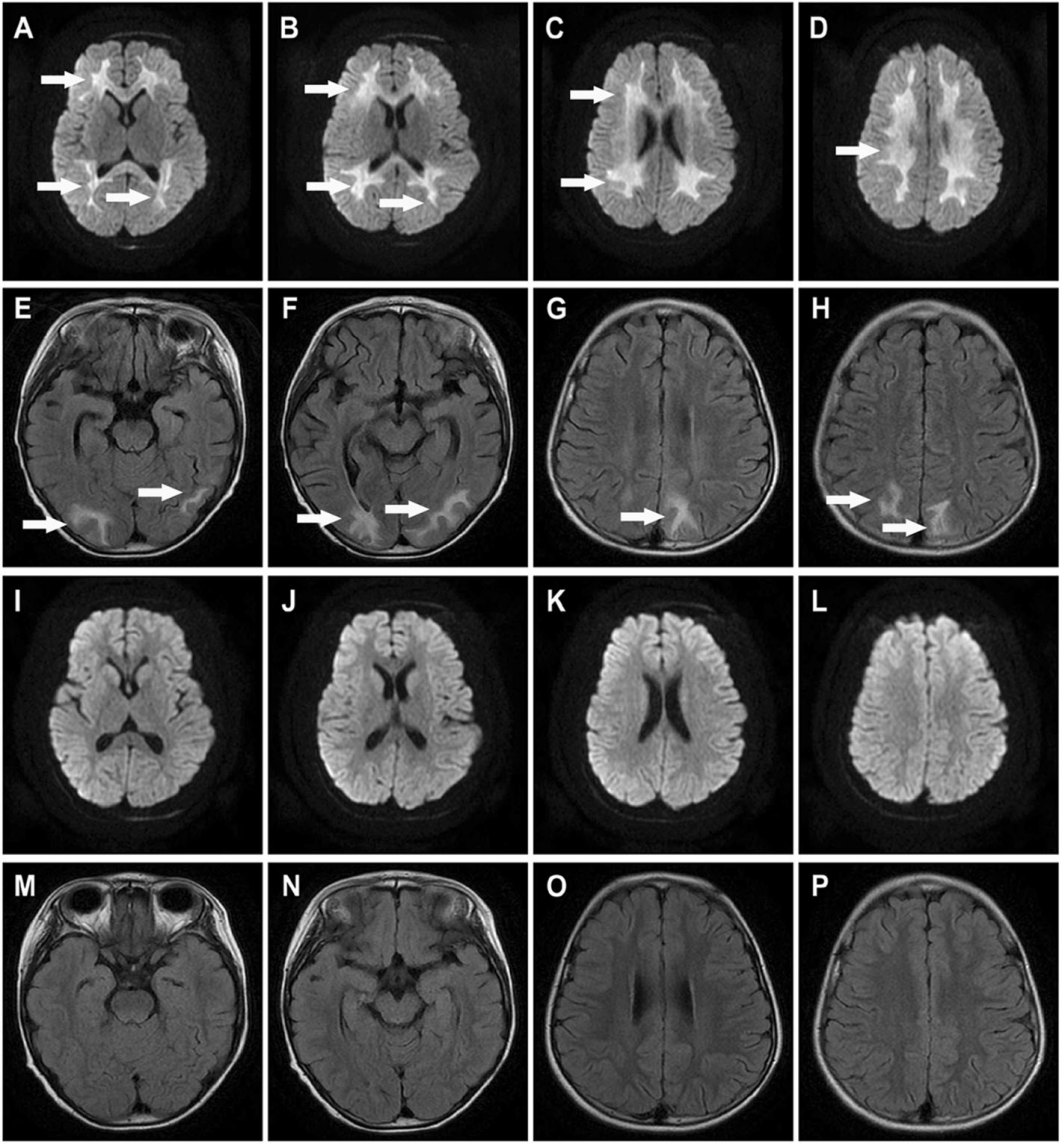
Brain magnetic resonance imaging (MRI) (**a-d**) DWI showed symmetric areas of high signal intensity in the periventricular white matter involving the centrum semiovale and corona radiate on day 1 (white arrow). (**e-h**) T2/FLAIR images showed diffuse high signal intensity in the white matter of occipital and parietal regions on day 17(white arrow). (**i-p**) DWI and T2/FLAIR images showed brain lesions had completely disappeared in the white matter on day 55

A previously healthy 5-year-old girl was admitted to the first hospital on January 17, 2018, with symptoms of abdominal pain, vomiting, and new-onset refractory status epilepticus (NORSE) that had lasted for 4 h. The results of a cranial computed tomography (CT) performed by the emergency department were normal. She was transferred to our hospital 6 h after onset, demonstrating signs of coma, tachypnea, and tachycardia. Her past medical history was negative. Upon admission, her body temperature was 36.3 °C, her heart rate was 155 beats/min, her respiratory rate was 50 breaths/min, her blood pressure was 102/67 mmHg, and the Glasgow Coma Scale score (GCS) was E1V1M3. Hypermyotonia was observed in both limbs, and the Babinski sign was positive bilaterally. Other findings from the systemic physical examinations were unremarkable. Percutaneous oxygen saturation measured by pulse oximetry (SpO2) was 93–95% on 25% FiO_2_. A series of blood tests showed the white blood cell (WBC) count to be 15.98 × 10^9^/L, neutrophils 91.3%, lymphocytes 4.8%, red blood cells 4.99 × 10^12^/L, platelets 264 × 10^9^/L, CRP 2.5 mg/L, procalcitonin (PCT) 55.77 ng/mL, ferritin 120 ng/ml, coagulant function (fibrin 1.53 g/L, D-Dimer 0.27 mg/L, prothrombin time (PT) 12.2 s, activated partial thromboplastin time (APTT) 24.9 s, INR 1.04, ACT 85%), serum enzymes (CKMB 24 U/L, CK 298 U/L, LDH 797 U/L, aspartate transaminase (AST) 65 IU/L, alanine transaminase (ALT), 38 IU/L), creatinine 40.7 μmol/L, and BUN 4.34 mmol/L. Brain magnetic resonance imaging (MRI) was performed quickly when the patient was admitted to our hospital. The diffusion-weighted imaging (DWI) scan of the patient’s brain on admission showed symmetric areas with high signal intensity in the periventricular white matter involving the centrum semiovale and the corona radiate (Fig. 1 a-d). The patient had rapid clinical deterioration that developed to hyperferritinemic sepsis with multiple organ dysfunction syndrome (MODS) 15 h after onset, including exhibiting a worsening mental status (GCS E1V1M1), fever (40.6 °C), hypotension (60/47 mmHg), tachycardia (heart rate: 178 beats/min), and tachypnea (respiratory rate: 70 breaths/min). She was intubated, and a vasopressor was quickly given to maintain blood pressure. A repeat blood test showed a WBC count of 9.09 × 10^9^/L, neutrophils 71.4%, lymphocytes 18.8%, red blood cells 4.49 × 10^12^/L, platelets 27 × 10^9^/L, ferritin 22,579.1 ng/ml, coagulant dysfunction (fibrin 1.5 g/L, D-dimer, 4.7 mg/L, PT 31.4 s, APTT 74.1 s, INR2.79, ACT 20.2%,), serum enzymes (CKMB 55 U/L, CK 1529 U/L, LDH 2550 U/L, AST 295 IU/L, ALT 114 IU/L), creatinine 134.3 μmol/L, and BUN 10.82 mmol/L. Blood cultures did not identify any pathogens. Serum viral studies (influenza A and B viruses, respiratory syncytial virus, adenoviruses, cytomegalovirus, Epstein–Barr virus, hepatitis C virus, hepatitis B virus, and human immunodeficiency virus) and serology tests for syphilis, Mycoplasma pneumonia and *Mycobacterium tuberculosis* were all negative. Lumber punctures were performed 3 times, and showed increased intracranial pressure (the highest was over 300 mm H_2_O). Cerebrospinal fluid (CSF) tests revealed that protein levels and glucose levels were normal without pleocytosis. CSF PCRs for enterovirus, herpes simplex virus, cytomegalovirus, Epstein-Barr virus, and tuberculosis were all negative. CSF bacterial and fungal cultures were also negative. The echocardiographic results showed that the left ventricular ejection fraction was 52%. The electrocardiograph (ECG) showed tachycardia. The electroencephalography examination demonstrated diffuse, generalized and slow background activity. X-ray imaging showed the presence of bronchitis. Hyperferritinemic sepsis was diagnosed after ruling out haemophagocytic lymphohistiocytosis (HLH) since the diagnostic criteria for HLH were not fulfilled in this girl. SAE was also diagnosed by ruling out encephalitis, meningitis, acute necrotizing encephalopathy, acute disseminated encephalomyelitis, Guillain-Barre syndrome, cerebral vasculitis, and metabolic encephalopathy, according to laboratory tests and imaging features. Integrated treatment was initiated on admission, which included anti-infection treatments (meropenem, vancomycin, and voriconazole), anti-inflammation treatments (methylprednisolone, 15 mg/kg/day × 3 days), intravenous immunoglobulin (1 g/kg/day × 2 days), and frozen plasma as well as the administration of other medicines for hepatic and myocardial protection. The symptoms were not relieved, and the fulminant development of MODS indicated that hyperinflammation or an autoimmune response might be involved. Membrane-based therapeutic plasma exchange (TPE) was started on day 3 since it can eliminate pro-inflammatory cytokines rapidly and can modulate the sepsis cascade. The exchange volume was 1.5-fold the patient’s plasma volume. Plasma volume was estimated as follows: plasma volume (in liters) = 0.07 × weight (kg) × (1-hematocrit) [3]. The removed plasma was substituted with fresh frozen plasma at a 1:1 ratio in our patient. The first three TPEs were performed daily. After the second TPE, the patient needed less vasopressor. By the third TPE treatment, liver enzymes had decreased. The patient was extubated and regained full consciousness on day 10, with mild remaining disseminated intravascular coagulation (DIC), acute kidney injury (AKI) and capillary leak syndrome (CLS). The last two TPEs were followed by continuous vena-venous hemofiltration (CVVH) 2 times to remove creatinine and BUN, to prevent liquid overload and to maintain coagulation function. After these integrated treatments, clinical and laboratory improvement were monitored in the patient (Table 1). Echocardiography showed that left ventricular systolic function was normalized, with a left ventricular ejection fraction (LVEF) of 62%. The ECG was normal. Her muscle strength gradually improved, showing increased movements, and she was able to walk short distances 17 days after onset. At this point, repeated brain MRI showed that the lesions were reduced, and T2/FLAIR scans indicated diffuse high signal intensity in the white matter of the occipital and parietal regions (Fig. 1 e-h). The patient was transferred to a rehabilitation center 21 days after onset. She had regained full independence in activities of daily living at 55 days after onset. A repeat brain MRI showed that the lesions had completely disappeared in the white matter regions (Fig. 1 i-p).

**Table 1:** Laboratory parameters for the patient

	Pre TPE	status post one TPE	status post two TPE	status post three TPE	status post last TPE
Hospital day	1	3	4	10	13
WBC(×10^9^/L)	9.09	8.67	8.16	5.7	11.28
neutrophil(×10^9^/L)	6.49	7.68	7.15	5.22	10.1
Platelet(×10^9^/L)	27	37	73	30	151
CRP (mg/L)	2.5	2.5	2.5	–	2.5
PCT (ng/mL)	55.77	–	21.82	20.54	0.21
Ferritin (ng/mL)	22,579.1	5246.03	–	1238.96	–
APTT(s)	74.1	43.3	31.5	34.8	31.7
PT(s)	31.4	23.5	18.1	20.5	12.8
Fib(g/L)	1.5	1.39	2.01	1.25	1.31
D-dimer (mg/L)	4.7	34.72	12.78	28.16	13.51
Creatine (μmol/L)	134.3	96.8	159.1	213.6	68.4
BUN (mmol/L)	10.82	9.03	12.99	23.29	10.31
ALT(U/L)	114	422	2580	1686	212
AST(U/L)	295	667	3415	1764	71
CKMB(U/L)	55	48	–	28	18

## Discussion and conclusions

MRI imaging finding on day 1 after onset of hyperferritinemic sepsis with SAE are rarely reported in pediatric patients. However, MRI could show abnormal lesion in the brain during the early course of SAE, which might contribute to appropriate management and therefore improve the prognosis of sepsis. The patient in our study presented with NORSE even though she was administered diazepam and phenobarbital. The symptom of NORSE last hours in our patient which indicated long-term consequences (after time point t_2_), including neuronal death, neuronal injury, and impaired consciousness [4, 5]. As expected, the imaging of MRI indicated diffuse lesion in her brain. It is very important to recognized NORSE quickly and require immediate interventions. NORSE is etiologically heterogeneous which may occur in patients with various encephalitis/encephalopathy, such as infectious encephalitis/encephalopathy, autoimmune encephalitis, and metabolic encephalopathy. Therefore, looking for the underlying causes can contribute to treatment correspondingly. NORSE in SAE is not rare. SAE can be found in up to 70–80% of patients with sepsis in intensive care units and can be an independent determinant for a poor prognosis [1, 6, 7]. There was a study that indicated that SAE can be an early feature of infection in the body and that it might appear before other systemic features of sepsis are obvious [8]. Tachypnea can be a feature of early SAE. Therefore, SAE can be latent but fatal in some patients; thus, distinguishing SAE early is very important for these patients. No specific biomarker exists for SAE, and it remains largely a clinical diagnosis [2, 9]. The blood tests showed that the WBC count, the proportion of neutrophils and PCT were very high in our patient which indicated the existence of bacterial infection. In particular, PCT was 55.77 ng/mL, which was helpful to differentiate system inflammatory reaction syndrome due to virus or autoimmune disease from bacterial sepsis. Furthermore, the amino acids and acylcarnitine results in the blood and urine organic acids excluded genetic metabolic diseases. Plasma ammonia was normal in this patient, including test for tetramines, organophosphorus pesticides, and sedatives, which were all negative in this patient. The patient did not show symptoms of skin rash or arthralgia. The antinuclear antibody spectrum was negative. There was no sign of autoimmune diseases, such as juvenile idiopathic arthritis, systemic lupus erythematosus, or vasculitis. The symptoms of this patient included fever, ferritin > 500 ng/mL, hypofibrinogenemia, and hemophagocytosis. These did not fulfill five of the eight criteria for HLH. Therefore, hyperferritinemic sepsis associated with MODS was diagnosed in our patient. We did not detect the autoimmune antibodies associated autoimmune encephalitis in this patient since she showed obvious symptoms of fulminant sepsis with MODS without outstanding symptoms of behavior, psychosis, or memory impairment. Additionally, the hyperferritinemia and high levels of PCT are rare in patients with autoimmune encephalitis.

A few pediatric cases of SAE have been reported, but they had poor prognoses. Neuroimaging in these patients with SAE was anfractuous. In this study, we collected data from 18 available cases of SAE (including 3 pediatric cases and 15 adult cases) in the literature, and the MRI features and prognoses are shown in Table 2. The data suggested that MRI results can be normal, or they can show multiple ischemic strokes, multiple microbleeds or white matter lesions in the periventricular areas and centrum semiovale in patients with SAE. The severity of lesions in the brain is positively correlated with the severity of sepsis, while it is inversely correlated with GCS scores [8]. The diffuse and severe white matter abnormalities in our case were rare and have been reported to be associated with poor outcomes in other patients with extensive white matter lesions [12]. Fortunately, MRI was performed during the early course of SAE in our case, which contributed to a favorable prognosis. However, apparent diffusion coefficient (ADC) mapping, which can mark geometric tissue characteristics such as the size and shape of the cell structure, is not performed routinely in our hospital. This is a limitation in our case. In fact, ADC mapping is very useful in the consideration of the pathogenesis of brain lesions which show a reduction in acute neurological diseases, but elevations in acute vasogenic edema formation or chronic tissue destruction [14].

**Table 2:** MRI features and prognosis of the patients with SAE in the literatures

Patient No.(reference)	Age/sex	Days from sepsis onset to MRI	MRI lesion	Outcome
1 [10]	2-years-and-nine-months-old/female	2	restricted diffusion in the basal ganglia and the subcortical white matter of the frontal and occipital lobes.	Brain death on day 73
2 [10]	17-month-old/man	106	cracking lesions in the white matter, brainstem and cerebellum.	Severe disability one and a half years later
3 [11]	4-year-old/female	3	diffuse brain edema with extended involvement of cortical and basal ganglia.	Recovery one year later
4 [12]	66-year-old/man	15	white matter hyperintensities and multiple microbleeds	Severe disability on day 60
5 [6]	53-year-old/female	28	a small number of white matter lesions	Moderate disability 7 month later
6 [13]	63-year-old / female	27	Diffuse white matter hyperintensities	Dead
7 [13]	82-year-old / female	8	Diffuse white matter hyperintensities and ischemic lesion	Survival on day 100
8 [13]	73-year-old /man	7	None of white matter hyperintensities and ischemic lesion	Survival on day 100
9 [13]	57-year-old /man	7	None of white matter hyperintensities and ischemic lesion	Survival on day 100
10 [13]	55-year-old /man	9	Pathch/cinfluent white matter hyperintensities	Survival on day 100
11 [13]	80-year-old / female	9	Punctiform white matter hyperintensities and ischemic lesion	Dead
12 [13]	44-year-old /man	12	None of white matter hyperintensities and ischemic lesion	Survival on day 100
13 [13]	76-year-old / female	4	Punctiform white matter hyperintensities	Dead
14 [13]	74-year-old / female	10	Pathch/cinfluent white matter hyperintensities	Survival on day 100
15 [13]	75-year-old /man	10	Diffuse white matter hyperintensities	Survival on day 100
16 [13]	54-year-old / female	17	None of white matter hyperintensities and ischemic lesion	Survival on day 100
17 [13]	60-year-old / female	4	Punctiform white matter hyperintensities	Survival on day 100
18 [13]	81-year-old /man	5	Pathch/cinfluent white matter hyperintensities and ischemic lesion	Survival on day 100

The treatment of SAE focuses on the appropriate management of sepsis, as SAE is not a direct infection in the CNS [8]. SAE can occur before sepsis is diagnosed. Thus, it is important to perform early investigations of and treatment for infection, thereby avoiding the development of severe SAE. Broad-spectrum antibiotics, fluid resuscitation, and organ support are important for treating sepsis [15]. Extracorporeal blood purification therapies might improve clinical outcomes for patients with sepsis by removing inflammatory mediators and bacterial toxins from circulation and by restoring immune function at the cellular level [16–18]. The child in our study presented with fulminant clinical features and extended brain lesions that indicated severe SAE. Thus, TPE was implemented for our patient. The extensive white matter lesions in SAE can be reversed, which has been reported in a few cases. However, the lesions in our patient receded faster than those observed in these cases, and the clinical outcome was good upon accurate early diagnosis and effective treatment in our patient.

In conclusion, the case we describe demonstrates a rare presentation of brain MRI in a pediatric patient with hyperferritinemic sepsis within the first days after the onset of symptom. SAE can be a fatal complication of sepsis that requires immediate diagnosis and treatment. Therefore, MRI should be performed early to diagnose this fatal complication and could play important roles in the improvement of the clinical outcomes by early initiation with appropriate treatments. However, it is necessary to further accumulate patients with SAE to confirm that the utility of MRI, especially ADC, would be useful to construct a systematic treatment protocol for similar patients in worldwide in the future.

## Data Availability

All primary data supporting the findings of this study are available within this article.

## Abbreviations

ADC: Apparent diffusion coefficient
AKI: Acute kidney injury
CLS: Capillary leak syndrome
CNS: Central nervous system
CRRT: Continuous Renal Replacement Therapy
CSF: Cerebrospinal fluid
CT: Computerized tomography
CVVH: Continuous vena-venous hemofiltration
DIC: Disseminated intravascular coagulation
DWI: Diffusion-weighted imaging
ECG: Electrocardiograph
GCS: Glasgow coma scale score
HLH: Haemophagocytic lymphohistiocytosis
LVEF: Left ventricular ejection fraction
MODS: Multiple organ dysfunction syndrome
MRI: Magnetic resonance imaging
NORSE: New-onset refractory status epilepticus
PCT: Procalcitonin
SAE: Sepsis-associated encephalopathy
TPE: Therapeutic plasma exchange
WBC: White blood cell
